# Development of the HT&Me intervention to support women with breast cancer to adhere to adjuvant endocrine therapy and improve quality of life

**DOI:** 10.1016/j.breast.2023.05.007

**Published:** 2023-05-27

**Authors:** Sarah-Jane F. Stewart, Joanna Slodkowska-Barabasz, Lucy McGeagh, Zoe Moon, Jo Brett, Mary Wells, Morven C. Brown, Mark Turner, Robert Horne, Deborah Fenlon, Farah Rehman, Henry Cain, Peter Donnelly, Victoria Harmer, Lesley Turner, Jan Rose, Linda Sharp, Eila Watson, the SWEET Research Team

**Affiliations:** a Research Department of Practice and Policy, UCL School of Pharmacy, University College London, UK; b Oxford Institute of Nursing, Midwifery and Allied Health Research, Oxford Brookes University, UK; c Imperial College Healthcare NHS Trust, UK; d Population Health Sciences Institute, Newcastle University, UK; e Newcastle University Centre for Cancer, Newcastle University, UK; f Research Software Engineering, Newcastle University, UK; g Faculty of Medicine and Life Science, Swansea University, UK; h Newcastle Upon Tyne Hospitals NHS Foundation Trust, UK; i South Devon Healthcare NHS Foundation Trust, UK; j Patient and Public Involvement Representatives, UK

## Abstract

**Background:**

Breast cancer is the most common cancer in women worldwide. Approximately 80% of breast cancers are oestrogen receptor positive (ER+). Patients treated surgically are usually recommended adjuvant endocrine therapy (AET) for 5–10 years. AET significantly reduces recurrence, but up to 50% of women do not take it as prescribed.

**Objective:**

To co-design and develop an intervention to support AET adherence and improve health-related quality-of-life (QoL) in women with breast cancer.

**Methods:**

Design and development of the HT&Me intervention took a person-based approach and was guided by the Medical Research Council framework for complex interventions, based on evidence and underpinned by theory. Literature reviews, behavioural analysis, and extensive key stakeholder involvement informed ‘guiding principles’ and the intervention logic model. Using co-design principles, a prototype intervention was developed and refined.

**Results:**

The blended tailored HT&Me intervention supports women to self-manage their AET. It comprises initial and follow-up consultations with a trained nurse, supported with an animation video, a web-app and ongoing motivational ‘nudge’ messages. It addresses perceptual (e.g. doubts about necessity, treatment concerns) and practical (e.g. forgetting) barriers to adherence and provides information, support and behaviour change techniques to improve QoL. Iterative patient feedback maximised feasibility, acceptability, and likelihood of maintaining adherence; health professional feedback maximised likelihood of scalability.

**Conclusions:**

HT&Me has been systematically and rigorously developed to promote AET adherence and improve QoL, and is complemented with a logic model documenting hypothesized mechanisms of action. An ongoing feasibility trial will inform a future randomised control trial of effectiveness and cost-effectiveness.

## Highlights

• Adjuvant endocrine therapy (AET) significantly reduces breast cancer recurrence.
• Up to 50% of women do not take AET as prescribed.
• The HT&Me intervention was designed to improve adherence to AET and quality of life.
• The HT&Me intervention appears acceptable and engaging to patients.

## 1 Introduction

Breast cancer is the most commonly diagnosed cancer worldwide, with around 2.3 million new cases and 685,000 deaths in 2020 [1]. Approximately 80% of breast cancers are oestrogen receptor positive (ER+). These patients are usually recommended oral adjuvant endocrine therapy (AET) such as tamoxifen or aromatase inhibitors (e.g. letrozole). AET significantly reduces the risk of recurrence and breast cancer death when taken for at least five years, with longer treatment now recommended for some patient groups with absolute reductions in recurrence of around 3–5% [[2], [3], [4], [5]].

Despite strong evidence of effectiveness, many women do not take AET as recommended. Up to 40% display suboptimal implementation [[6], [7], [8], [9], [10], [11], [12], [13]] and up to 20% stop taking AET completely within two years, rising to 50% by five years [6,8,10,11]. Determinants of poor AET adherence are complex and can reflect condition-related (e.g., co-morbidities), medication-related (e.g., side-effects), socio-economic-related (e.g., poorer social support), health-care system-related (e.g., poorer relationships with health professionals), and patient-related (e.g., negative AET beliefs) factors [6,11,[14], [15], [16], [17]]. Moreover, commonly reported side-effects such as hot flushes and arthralgia impact quality-of-life (QoL) as well as adherence [18,19].

Interventions to improve AET adherence have largely been ineffective [[20], [21], [22], [23]]. Most have focussed on education, which is insufficient to change behaviour [[24], [25], [26]], with a more multifaceted approach likely required. Guidance for developing complex interventions notes the importance of basing an intervention on a comprehensive understanding of determinants of the target behaviour (adherence), relevant theory, and involving users [[27], [28], [29]]. Developers should also consider how their intervention would be expected to change behaviour (i.e. mechanisms of action). The extent to which these requirements were met in previous interventions is not clearly specified.

Self-management refers to someone's ability to manage the symptoms, treatment, physical and psychosocial consequences and lifestyle changes associated with living with chronic conditions. Accumulating evidence shows the benefits of self-management support in empowering individuals living with chronic conditions [[30], [31], [32], [33]]. In cancer, self-management is associated with reductions in symptom severity and improvements in self-efficacy and QoL [[34], [35], [36]]. Partnering with healthcare providers is key for developing core skills for self-management [31]. There is also growing interest in the potential of e-health/m-health digital interventions in supporting self-management [37,38]. Such interventions offer advantages of scalability and low-cost delivery, although potentially at lower efficacy than interventions led by a trained professional and with increased risk of attrition and exclusion. Recognising the value that patients place on personal support [39], a blended approach, combining digital tools with healthcare professional (HCP) interaction could be effective.

This paper reports on the development and optimisation of the HT&Me intervention, as part of the SWEET (Supporting Women with adhErence to hormonE Therapy following breast cancer) research programme (https://fundingawards.nihr.ac.uk/award/NIHR200098). HT&Me is a blended supported self-management intervention which seeks to encourage adherence to AET and improve QoL in women with ER + breast cancer, while being scalable and implementable within the UK National Health Service (NHS). Here we describe: the intervention development process; components, content and mode of intervention delivery; and mechanisms through which it is expected to impact AET adherence and QoL.

## 2 Methods

### 2.1 Intervention design & development process

**Fig. 1:**
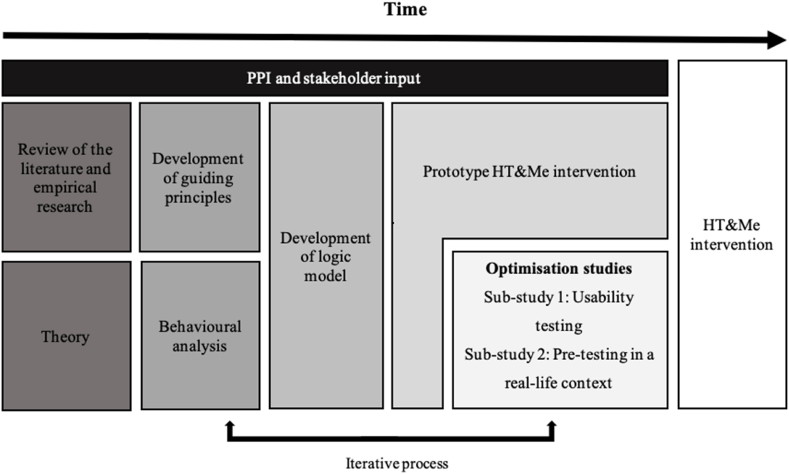
An overview of the HT&Me intervention development process.

Following the UK Medical Research Council (MRC) guidance on complex interventions [[27], [28], [29]], HT&Me was developed using a theoretically informed, evidence-based and person-centred approach, and reported with reference to the GUIDED [40] and TIDieR [41] guidance (Supplementary Material 1, 2). Fig. 1 presents an overview of the process.

The multidisciplinary core development team included an epidemiologist, health service researchers, health psychologists and nurses with expertise in breast cancer care and medication adherence. This team met weekly to plan and draft intervention materials.

#### 2.1.1 Key stakeholder input

Service users were central to the process. Two patient and public involvement (PPI) representatives (women previously prescribed AET for breast cancer) were grant co-applicants, attended key meetings, and were involved in all core research decisions, including intervention development. A Patient Advisory Group (PAG) of 11 women prescribed AET was formed and actively involved throughout intervention development, including women who had discontinued AET. A Community of Interest (CoI) group of 28 women offered AET (including women who declined treatment) were recruited through charities and PPI networks and advised on a range of topics to inform intervention content (e.g. tips for side-effect management).

A Clinical Reference Group (CRG) comprising breast surgeons, oncologists, breast cancer nurses, pharmacists, GPs and a community nurse, met regularly throughout development to ensure the suitability of the intervention for the target population. This group also advised on optimal intervention delivery across different patient pathways and ways to overcome potential barriers to NHS implementation.

#### 2.1.2 Evidence review and behavioural analysis

Drawing upon our previous empirical work [6,[15], [16], [17],^42^] and systematic reviews [11,14], as well as additional published reviews, we conducted a behavioural analysis of determinants of AET adherence.

#### 2.1.3 Theory

The intervention followed the Perceptions and Practicalities Approach (PaPA) for supporting adherence. The PaPA is a pragmatic framework for designing adherence support, highlighting the importance of tailoring support to an individual's needs [43]. It draws on wider theoretical models emphasising the importance of patients' representations of illness and treatment, including Leventhal's Common Sense Model [44,45], and the Necessity Concerns Framework [46,47]. We have shown, in empirical work, that the PaPA is useful for understanding AET adherence [42,48], and it posits that to best support adherence, both perceptual and practical barriers underpinning an individual's motivation and ability to adhere to treatment should be addressed. Beliefs about how *necessary* a woman perceives taking AET to be (e.g., to reduce risk of recurrence), relative to their *concerns* about taking it (e.g., worries about AET side-effects) are particularly important [11]. PaPA is recommended by the English National Institute for Health & Care Excellence (NICE) guidelines and the National Co-ordinating Centre for NHS Service Delivery and Organisation [49,50].

#### 2.1.4 Development of guiding principles and intervention logic model

Guiding principles - which identify key design objectives and specific intervention features to address these objectives - were developed, drawing upon team expertise of facilitators and barriers to AET adherence, prior PPI work, and views of the PAG and CoI. A draft logic model [51] was developed to illustrate relationships between programme inputs, intervention-related activities and desired outcomes (improved adherence and QoL), and refined as development progressed.

#### 2.1.5 Online intervention design workshops

Stakeholder intervention development workshops were conducted online because of COVID-19 restrictions. Two workshops were completed with eight women prescribed AET for breast cancer, recruited via Independent Cancer Patients’ Voice (ICPV) and the newsletter/patient forum of the charity Breast Cancer Now (BCN). A further two workshops with breast nurses from three hospital Trusts were conducted online. Initial intervention ideas were generated and discussed. We developed these ideas with our CRG and breast cancer charity representatives (ICPV and BCN). We worked closely with our PAG through two-monthly online meetings, focussing on intervention content, and usability and visual acceptability of the digital elements. This process, coupled with learning from previous apps [52], led to the creation of the prototype HT&Me intervention.

### 2.2 Optimisation of the digital component of the HT&Me intervention

Whilst there are some potential limitations with the platform (e.g. no offline availability), a web-app was chosen to maximise flexibility to use across devices (e.g. mobile phones, tablets, laptops) and to minimise risk of long-term maintenance issues due to operating system updates. The user-interface was designed with careful consideration given to user demographics as well as design guidelines from gov. uk [53] and the NHS [54]. In discussions with the PAG, it was decided not to tunnel users through content sequentially, but grant access to all sections simultaneously, allowing the user to select according to their needs. The platform was tested on all major browsers and devices to ensure the widest possible access. It conforms to industry standard practices, and is hosted in the cloud using Microsoft Azure, thus permitting scale-up as required.

Two sequential qualitative studies collected feedback to optimise the HT&Me web-app. Ethical approval was gained from the London Central Ethics Committee (ref no. 21/PR/0603) and informed consent was given. Participants were entered into a prize draw (£50 gift voucher) as thanks for their participation. Participant characteristics are described in Supplementary Material 3. In *study 1* online think-aloud interviews tested acceptability and usability by gathering ‘live’ feedback to the HT&Me web-app from 20 women prescribed AET within the last three years, interviewed in “batches” of 3–5 women. Content analysis was undertaken [55] and feedback tabulated using the ‘Table of Changes’ (TOC) method. Modifications were made in line with our Guiding Principles and were prioritised based on the must-have, should-have, could-have, and won't-have (MoSCoW) criteria [56], then the next round of interviews was conducted. In *study 2* semi-structured interviews explored experiences from participants who independently used the HT&Me web-app for 2–3 weeks prior to interview. Fifteen participants with breast cancer, first prescribed AET within the past 12-months were recruited through three NHS Trusts. As for sub-study 1, interviews were conducted in batches (approx. N = 3), content analysis was undertaken53, and prioritised modifications to the HT&Me web-app were made.

## 3 Results

### 3.1 Behavioural analysis

Table 1 summarises the key determinants of AET adherence identified in our behavioural analysis. The analysis also identified potential barriers and facilitators to intervention engagement, and target behaviours relating to adherence and QoL. The identified behaviours and their determinants were mapped onto the Behaviour Change Technique (BCT) Taxonomy [56,57], and the PaPA to identify postulated mechanisms of action (Table 2). Intervention content was then designed to address each barrier and facilitator, using the relevant BCTs.

**Table 1:** Determinants of AET adherence to be addressed in the intervention.

Perceptions/experience	Practicalities/enablers	Other factors
Beliefs about breast cancer[11,17,42]	Behavioural regulation (e.g. habit formation)[6,14]	Other cancer-directed treatments[11]
Beliefs about AET treatment[6,11,[14], [15], [16], [17],^42^]	Knowledge about AET[14,15,17]	Clinical factors[6,11,42]
Outcome expectancies/beliefs about consequences (e.g., taking/not taking AET)[6,11,[14], [15], [16], [17],^42^]	Social support[6,11,14,17,42]	
Attitudes towards behaviour (adherence)[16,17]	Emotional regulation[6,14,15,42]	
Self-efficacy (to take AET)[11,14,42]	Support from HCPs[11,[14], [15], [16], [17],^42^]	
Social influences[6,11,14,17,42]		
Goals and motivation[6,15]		
Emotion[6,11,14,42]		
AET is ongoing reminder of cancer[15]		
Bother/impact of AET side-effects (& coping strategies)[6,11,[14], [15], [16], [17],^42^]		
Trust in HCPs[11,15,17]

**Table 2:** An overview of HT&Me intervention content, mechanisms of actions and behavioural change techniques (BCTs).

Intervention component	Content	Mechanisms of action	BCTs: Adherence	BCTs: for behaviours indirectly targeting to adherence and QoL
**HT&Me animation video ‘Understanding Hormone Therapy’**	A short animation video explaining what AET is, how it works, and the importance of taking it every day	• Increase knowledge about AET • Increase necessity beliefs • Reduce concerns • Remove practical barriers to taking AET	1.4 Action Planning 3.1 Social Support (unspecified) 5.1 Information about Health Consequences 8.3 Habit formation	
**Study Nurse consultations**	Two consultations facilitated by HT&Me Study Nurses to: • Address perceptual (e.g. doubts about necessity, concerns about treatment) and practical (e.g. forgetting) barriers to AET adherence. • Introduce women to the HT&Me web-app • Provide tailored information and support to address and overcome any identified barriers, signposting to relevant sections of the HT&Me web-app	• Increase knowledge about AET • Increase necessity beliefs • Reduce concerns • Address beliefs about consequences of not taking AET • Empower women to know where they can seek support	5.1 Information about health consequences 8.3 Habit formation 15.1 Verbal persuasion about capability	3.1 Social support (unspecified) 3.2 Social support (practical)
***HT&Me webapp:***				
**Core informational based HT&Me web-app sections**				
**Taking Hormone Therapy**	• The HT&Me animation video • Information about how AET helps to keep cancer from coming back • Questions and answers about AET in general and the risks and benefits of AET • Information about combining AET with other breast cancer treatments • Links to other useful resources and relevant websites • A facility to make a plan to take AET every day	• Increase knowledge about AET • Increase necessity beliefs • Reduce concerns • Address beliefs about consequences of not taking AET • Behavioural regulation (e.g. habit formation) • Remove practical barriers to taking AET • Increase self-efficacy for taking AET	1.1 Goal setting (behaviour) 1.4 Action Planning 4.2 Information about antecedents 5.1 Information about Health Consequences 7.1 Prompts/cues 8.3 Habit formation 9.1 Credible source 15.1 Verbal persuasion about capability	
**Dealing with Side-effects**	• Information about and practical hints and tips to support the self-management of the most common side-effects of hormone therapy: hot flushes, joint aches and pains, fatigue, sexual concerns, weight changes, mood changes, vaginal dryness and pain, sleep problems and other side-effects (problems with memory, feeling sick, skin changes, headaches) • Cognitive Behavioural Therapy (CBT) style thought reframing activity (‘My Thoughts’) to develop effective coping with distressing side-effects • Links to additional resources and relevant websites • Advice on when to seek further help	• Reduce concerns about AET • Provide coping strategies for side-effects • Increase confidence in managing side-effects • Reduce impact of side-effects • Improve QoL	15.1 Verbal persuasion about capability 4.3 Re-attribution	1.2 Problem solving 2.3 Self-monitoring of behaviour 2.4 Self-monitoring of outcome(s) of behaviour 3.1 Social Support (unspecified) 3.2 Social support (practical) 3.3 Social support (emotional) 4.2 Information about antecedents 4.4 Behavioural experiments 7.1 Prompts/cues 9.1 Credible source 11.2 Reduce negative emotions 12.1 Restructuring the physical environment 12.6 Body changes 13.2 Framing/reframing 15.3 Focus on past success 15.4 Self-talk
**Healthy Living, Healthy Mind**	• ‘Information and advice on making lifestyle changes tailored towards women taking AET (‘Being active’, ‘Healthy Eating’ and ‘Quitting Smoking’). • Goal setting for ‘Being Active’ and ‘Healthy Eating’ • Information and tips to support mental health and wellbeing, by addressing topics such as fear of recurrence (‘Dealing with the Emotional Impact of Cancer’). • Links to additional resources and relevant websites	• Improve physical activity/diet • Reduce impact of side-effects • Reduce emotional distress associated with cancer • Improve QoL	5.1 Information about health consequences	1.3 Goal setting 1.2 Problem solving 1.4 Action planning 1.7 Review behaviour goal 5.1 Information about health consequences 3.1 Social support (unspecified) 3.2 Social support (practical) 3.3 Social support (emotional) 9.1 Credible source 11.2 Reduce negative emotions 12.4 Distraction 13.2 Framing/reframing 15.1 Verbal persuasion about capability
**Help & Support**	• Information on what support is available, and hints and tips for getting support from and talking to others including their breast cancer team, friends, and family. • Links to additional sources of support (helplines, forums, websites etc.)	• Reduce concerns • Remove practical barriers to taking AET • Empower women to know where they can seek support • Improve social support • Improve relationship with HCPs		3.1 Social support (unspecified) 3.2 Social support (practical) 3.3 Social support (emotional) 9.1 Credible source
**Interactive sections of HT&Me web-app**				
**My Personal Support**	Women are prompted to complete ‘My Personal Support’ upon logging into the HT&Me web-app for the first time, but participants can go into this section and complete it again as their beliefs and experiences change. It offers tailored informational support by profiling key perceptual and practical barriers to taking AET based on participants answers to questions about: i) How necessary they perceive taking AET to be ii) Any concerns they have about taking AET iii) Any practical barriers impacting on their ability to take AET as prescribed	• Reduce forgettingRemove practical barriers to taking AET • Increase self-efficacy for taking AET • Increase confidence in managing side-effects • Reduce concerns about AET • Increase knowledge about AET • Increase necessity beliefs • Address beliefs about consequences of not taking AET • Behavioural regulation (e.g., habit formation)	2.3 Self-monitoring of behaviour 2.4 Self-monitoring of outcome(s) of behaviour 7.1 Prompts/cues	2.3 Self-monitoring of behaviour 2.4 Self-monitoring of outcome(s) of behaviour 7.1 Prompts/cues
**My Hormone Therapy Diary**	• Record side-effects and view them on a graph • Record taking hormone therapy tablet • Set text or email reminders to take AET and collect prescriptions	• Reduce forgetting • Increase confidence in managing side-effects • Behavioural regulation (e.g., habit formation) • Remove practical barriers to taking AET • Increase self-efficacy for taking AET	1.1 Goal setting 1.4 Action planning 3.1 Social support (unspecified) 5.1 Information about health consequences 7.1 Prompts/cues	3.1 Social support (unspecified)
**My Goals and Plans**	• View, review or set physical activity or healthy eating goals • View or edit a plan to take AET • View or edit ‘My Thoughts’ activities	• Reduce impact of side-effects • Improve QoL • Increase self-efficacy for taking AET • Behavioural regulation (e.g., habit formation)	1.1 Goal setting 1.2 Problem solving 1.4 Action planning 1.5 Review behaviour goal(s) 8.3 Habit formation	1.1 Goal setting 1.2 Problem solving 1.4 Action planning 1.5 Review behaviour goal(s) 11.2 Reduce negative emotions 13.2 Framing/reframing
**Text/email ‘nudge’ messages**	At regular intervals women are sent two types of ‘nudge’ messages via email or text message according to individual preference to i) prompt adherence, reinforce the importance of continuing therapy, and indicate support is available if needed via the web-app ii) encourage women to visit the *My Personal Support* section of the web-app to access tailored information and support	Directly: • Increase knowledge about AET • Increase necessity beliefs Indirectly (via signposting back to HT&Me web-app) • Reduce concerns • Remove practical barriers to taking AET	1.4 Action planning 7.1 Prompts/cues 8.3 Habit formation	1.2 Problem solving

### 3.2 Guiding principles and logic model of change

**Fig. 2:**
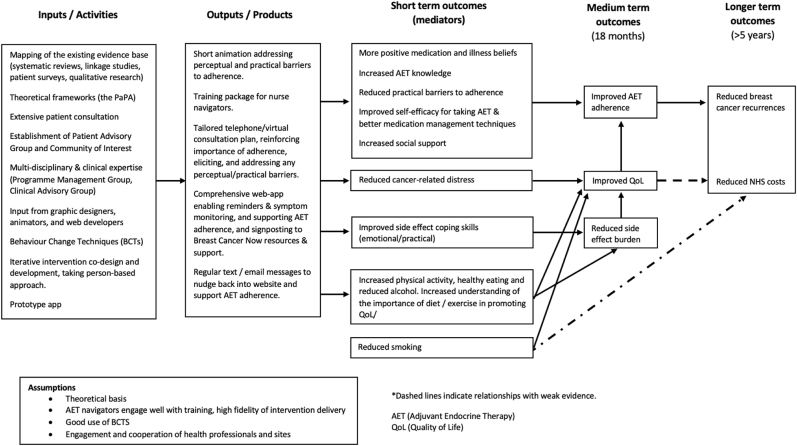
The HT&Me logic model of change.

The guiding principles are presented in Table 3. The HT&Me logic model and mechanisms of action through which we expect HT&Me to improve adherence and QoL, and ultimately to reduce breast cancer recurrence and NHS costs, is shown in Fig. 2. A no-blame approach was taken throughout, understanding that for some women, non-adherence may be an informed choice.

**Table 3:** The HT&Me intervention: Guiding principles.

Key findings from the literature	Intervention design objective	Key Intervention feature(s) to address design objective
Good evidence that beliefs doubting the necessity of AET and high concerns about taking AET may contribute to low adherence to AET.	Encourage women to see AET as an integral part of their breast cancer treatment.	HT&Me web-app to inform about the importance of AET in terms of reducing risk of recurrence by i) increasing beliefs that AET is Necessary and ii) reducing AET-related Concerns.
A ‘no-blame’ approach is a key component of the PaPA. Patients are often wary of disclosing nonadherence for fears of being judged or being branded a ‘bad patient’ [43]. Tailored adherence interventions are more effective than non-tailored interventions [43].	Adopt a person-centred, supportive and tailored approach which is not judgemental, patronising or over medicalised.	Tailored content addressing necessity/concerns beliefs via ‘My Personal Support’ in HT&Me web-app and nurse consultation. New information/education presented in a way that acknowledges the existing beliefs patients may hold about AET. Recognise importance of fully informed choice.
Good quality outcomes of AET depend on optimal self-management by the patient [[58], [59], [60]].	Support women to self-manage their AET and any side-effects (whilst recognising informed choice).	HT&Me web-app to provide information and evidence-based advice for the self-management of taking AET and associated side-effects. HT&Me web-app to include interactive and tailored elements such as reminders, a side-effect diary, and goal setting activities. Simple and clear web-app/materials layout. HT&Me and Study Nurse consultations to signpost to additional resources (helplines, websites, forums).
	Potentially scalable and implementable within the NHS	Ensuring the HT&Me intervention is flexible and adaptable to local contexts by focussing on the self-management of AET adherence and side-effects.

### 3.3 Design and refinement of the HT&Me intervention

The HT&Me intervention consists of four elements: i) an animation video, ii) nurse consultations, iii) web-app and iv) regular ‘nudge’ messages (Supplementary Material 4). Table 3 provides an overview of content and BCTs directly targeting the core behaviour of adherence, and behaviours indirectly targeting adherence and QoL, such as healthy eating and physical activity.

#### 3.3.1 Animation video

Women's first engagement with HT&Me is via a short (6 min) animation video, provided as a weblink soon after AET initiation. The script was written by the research team with CRG and PAG input. A storyboard was created by the designers and reviewed by the core development team and PAG, who inputted on key concepts such as how to visually represent how the treatment works and why it is important to take it each day. This animation is also housed within the HT&Me web-app.

#### 3.3.2 Nurse consultations

PPI input and the evidence review indicated that contact with HCPs was important to women. This was integrated in the form of an initial and follow-up consultation with a Study Nurse (a professional with experience of working with women with breast cancer). The initial 30 min consultation (either face-to-face or remote via video conferencing software) follows the PaPA approach to supporting adherence [43]. It is tailored to each individual, elicits and addresses women's beliefs about the necessity of taking AET, concerns they have about taking it, and any practical difficulties with taking it (e.g. forgetting). The nurse introduces women to the HT&Me web-app and signposts relevant sections of the web-app.

Approximately 3 months later, women are offered a 15–20 min telephone or video-call follow-up consultation. This appointment communicates the continuing importance of treatment and seeks to address any emerging AET-related concerns or issues and re-directs them to the web-app where necessary.

The Study Nurses delivering the intervention are trained via a self-directed five-module online training course (approx. 7hrs) developed by the study team, with CRG input, comprising videos and interactive activities to provide an understanding of the theoretical rationale underpinning the intervention, a detailed overview of intervention components and procedures, and examples of a typical consultation (Table 4). Study Nurses also receive consultation guides which include key discussion points, and possible questions and answers. A proportion of consultations will be recorded and reviewed by the research team to assess fidelity.

**Table 4:** An overview of the HT&Me Nurse Training.

Nurse Training component	Content
Module 1	An introduction to the SWEET programme and hormone therapy
Module 2	An introduction to the HT&Me web-app
Module 3	An introduction to the Perceptions and Practicalities Approach (PaPA) to supporting adherence and theconsultationguide
Module 4	A typicalconsultation
Module 5	Study paperwork and logistics

#### 3.3.3 Prototype web-app

The HT&Me web-app comprises four primarily knowledge-based sections (‘Taking Hormone Therapy’, ‘Dealing with Side-effects', ‘Healthy Living, Healthy Mind’ and ‘Help and Support’) (Supplementary Material 5). These provide information, hints and tips for taking AET and side-effect management, videos, activities, and real-life experiences from women with breast cancer. The web-app also includes three interactive components (‘My Hormone Therapy Diary’, ‘My Goals and Plans’ and ‘My Personal Support’). These enable women to make a plan for how they will take AET daily, set a text/email medication reminder, log experiences with taking AET, and monitor side-effects. Women can set healthy eating and physical activity goals and undertake activities targeted at addressing low mood. ‘My Personal Support’ is based on the PERSIGNIA™ system which applies the PaPA framework and comprises three components: (1) brief profiling tools (i.e. questions), adapted from validated questionnaires, assessing (a) doubts about personal need for AET, (b) concerns about AET, (c) practical difficulties with the treatment; (2) specific support (i.e information/messages and direction to relevant sections of the web-app for more detail) to address the specific adherence barriers identified under (a), (b) and (c) above; and (3) a proprietary algorithm linking specific support to specific barriers. It was important that the web-app was visually pleasing and so the help of a designer was enlisted.

#### 3.3.4 Motivational ‘nudge’ messages

Participants also receive monthly, brief, motivational ‘nudge’ messages, delivered by email or text (depending on preference), promoting adherence and encouraging use of the web-app in the event of questions or problems. In addition, every 6-months they receive a message encouraging them to complete ‘My Personal Support’.

#### 3.3.5 HT&Me web-app optimisation studies

In study 1 the prototype HT&Me web-app was generally well received. Participants found the language on the web-app clear and easy to understand and described the content as helpful. For example one participant said, *“I didn't expect it to go into such depth of explanation about what tamoxifen means […] that's brilliant how that was done.”* Several barriers to engagement were highlighted which prompted improvements in functionality, a more user-friendly interface and more inclusive content; for example, younger women and those from Black and Asian ethnic groups did not feel well represented so quotes and images were changed to be more inclusive. See Supplementary Material 6 for a breakdown of patient quotes and intervention amendments.

In study 2 participant feedback was generally very positive. Some women identified topics that were not covered by the web-app content, such as information about how AET differs to hormone replacement therapy and advice for the management of chills after experiencing hot flushes. For example, “*I had side-effects that weren't on there […] I get chills.”* This prompted us to add information on such topics to the web-app. See Supplementary Material 7 for a breakdown of patient quotes and intervention amendments.

Although most women who used the interactive features of the HT&Me web-app found them useful, these were reported to be used less frequently than expected. To increase engagement with these elements, modifications were made to the Study Nurse training and consultation guide. These encourage Study Nurses to emphasise the tailored nature and value of these features and highlight them as ‘key’ areas of the web-app to participants during their initial consultation when the web-app is introduced.

## 4 Discussion

This paper describes a theory-, evidence- and person-based approach to the development of HT&Me, a supported self-management intervention to encourage adherence to AET and improve QoL in women with ER + breast cancer.

We provide preliminary evidence that a blended, supported self-management intervention, is likely to be acceptable and engaging to patients. Participant feedback was used to identify potential barriers to patient engagement with the HT&Me web-app and to inform modifications to improve its acceptability and persuasiveness as a tool to encourage adherence to AET. Moreover, the involvement of a wide range of HCPs throughout maximises the chances that the intervention will be feasible to deliver in real-world clinical practice and scalable across the UK NHS.

HT&Me responds to calls for improved quality of supported self-management for those living with cancer [61]. Critically, it addresses each of the core skills for self-management of cancer as a chronic disease, outlined in Howell et al. (2021), namely problem solving, decision-making, behavioural self-monitoring and tailoring, setting goals and action planning, partnering with healthcare providers, risk reduction and health maintenance [31]. This paper explicitly describes the systematic processes involved in developing the HT&Me intervention, addressing the critique that lack of transparency in describing self-management interventions in cancer has so far inhibited implementation [62].

The approach outlined in this paper, and the HT&Me intervention itself, could also provide a framework for rigorous and systematic design and development of interventions to support adherence to other oral anti-cancer medicines and those for other long-term conditions, where concerns have been expressed about the implications of non-adherence for patient outcomes [[62], [63], [64]].

### 4.1 Limitations

Women who self-selected to take-part in the web-app optimisation studies may have been particularly interested in support for AET adherence andor AET adherence and therefore more likely to find the HT&Me web-app interesting and engaging. Despite efforts to recruit a diverse sample of women with suboptimal adherence to AET, many participants were adherent and white, meaning we cannot be entirely sure, at this stage, of acceptability and usefulness of the intervention to poorly adherent women and those of black and minority ethnic groups.

Women self-reported usage of the web-app and so may have overstated their use. Further, user feedback was collected after 2–3 weeks of use. It is possible this time window was not sufficient for women to fully experience using all elements of the web-app. Finally, we selected qualitative methods to be best suited for informing iterative refinements of the web-app. It is possible that use of a questionnaire, with a larger sample of women, may have yielded additional information.

## 5 Conclusions

HT&Me has been systematically and rigorously developed to promote AET adherence and improve QoL, and is supported with a logic model documenting hypothesized mechanisms of action. Preliminary data suggests the intervention is acceptable and engaging to patients. An ongoing single arm feasibility trial will inform a future randomised control trial of intervention effectiveness and cost-effectiveness.

## Funding

This article presents independent research funded by the 10.13039/501100000272National Institute for Health Research (NIHR) under the Programme Grants for Applied Research programme [NIHR200098]. The views expressed in this article are those of the authors and not necessarily those of the NHS, the NIHR or the Department of Health.

## Author contributions

LS, EW, MW, RH, DF, JB, JR, LT, PD obtained the funding for the work. The HT&Me intervention was conceptualised by EW, LS, LMG, JB, MW, RH, DF, FR, HC, PD, LT and JR. The content for HT&Me was created by LMG, SFS, ZM, JSB, JB, MW, RH, EW and LS, with clinical expertise and input from DF, FR, HC, PD and VH and patient input from LT & JR. MCB developed the protocol for the optimisation studies. Women were interviewed by JSB, SFS and ZM for the optimisation studies, and this data was analysed predominantly by JSB with input from SFS, ZM, EW and LS. MT led the web-app build. The manuscript was written by SFS, JSB, EW and LS. All authors critically reviewed the manuscript and approved the submitted version.

## Declaration of competing interest

VH reports private work conducted with Bioderma, Lilly, Medscape, Roche and Digistain. RH reports speaker engagement with honoraria for AbbVie, Abbott, Amgen, Astellas, AstraZeneca, Boehringer Ingelheim, Biogen, Gilead Sciences, GlaxoSmithKline, Janssen, Merck Sharp Dohme, Merck, Novartis, Pfizer, Procter & Gamble, Roche, Sanofi, Shire Pharmaceuticals, TEVA, UCB, personal consultancy for Amgen, Abbott, AstraZeneca and Novartis. He is the Founding Director of a UCL-Business company (Spoonful of Sugar Ltd) providing consultancy on treatment engagement and patient support programmes to healthcare policy makers, providers and pharmaceutical industry. ZM reports paid work for UCL Business Company Spoonful of Sugar Ltd.

## References

[1] Sung H., Ferlay J., Siegel R.L. (2021). Global cancer statistics 2020: GLOBOCAN estimates of incidence and mortality worldwide for 36 cancers in 185 countries. CA A Cancer J Clin.

[2] Dowsett M., Cuzick J., Ingle J. (2010). Meta-analysis of breast cancer outcomes in adjuvant trials of aromatase inhibitors versus tamoxifen. J Clin Oncol.

[3] Early Breast Cancer Trialists’ Collaborative Group (EBCTCG) (2015). Aromatase inhibitors versus tamoxifen in early breast cancer: patient-level meta-analysis of the randomised trials. Lancet.

[4] Davies C., Godwin J., Early Breast Cancer Trialists’ Collaborative Group (EBCTCG) (2011). Relevance of breast cancer hormone receptors and other factors to the efficacy of adjuvant tamoxifen: patient-level meta-analysis of randomised trials. Lancet.

[5] Qian X., Li Z., Ruan G., Tu C., Ding W. (2020). Efficacy and toxicity of extended aromatase inhibitors after adjuvant aromatase inhibitors-containing therapy for hormone-receptor-positive breast cancer: a literature-based meta-analysis of randomized trials. Breast Cancer Res Treat.

[6] Cahir C., Barron T.I., Sharp L., Bennett K. (2017). Can demographic, clinical and treatment-related factors available at hormonal therapy initiation predict non-persistence in women with stage I-III breast cancer?. Cancer Causes Control.

[7] Huiart L., Ferdynus C., Giorgi R. (2013). A meta-regression analysis of the available data on adherence to adjuvant hormonal therapy in breast cancer: summarizing the data for clinicians. Breast Cancer Res Treat.

[8] Huiart L., Ferdynus C., Giorgi R. (2013). Tamoxifen therapy for patients with breast cancer. Lancet.

[9] Makubate B., Donnan P.T., Dewar J.A., Thompson A.M., McCowan C. (2013). Cohort study of adherence to adjuvant endocrine therapy, breast cancer recurrence and mortality. Br J Cancer.

[10] McCowan C., Shearer J., Donnan P.T. (2008). Cohort study examining tamoxifen adherence and its relationship to mortality in women with breast cancer. Br J Cancer.

[11] Moon Z., Moss-Morris R., Hunter M.S., Carlisle S., Hughes L.D. (2017). Barriers and facilitators of adjuvant hormone therapy adherence and persistence in women with breast cancer: a systematic review. Patient Prefer Adherence.

[12] Murphy C.C., Bartholomew L.K., Carpentier M.Y., Bluethmann S.M., Vernon S.W. (2012). Adherence to adjuvant hormonal therapy among breast cancer survivors in clinical practice: a systematic review. Breast Cancer Res Treat.

[13] van Herk-Sukel M.P.P., van de Poll-Franse L.V., Voogd A.C., Nieuwenhuijzen G.A.P., Coebergh J.W.W., Herings R.M.C. (2010). Half of breast cancer patients discontinue tamoxifen and any endocrine treatment before the end of the recommended treatment period of 5 years: a population-based analysis. Breast Cancer Res Treat.

[14] Cahir C., Guinan E., Dombrowski S.U., Sharp L., Bennett K. (2015). Identifying the determinants of adjuvant hormonal therapy medication taking behaviour in women with stages I–III breast cancer: a systematic review and meta-analysis. Patient Educ Counsel.

[15] Moon Z., Moss-Morris R., Hunter M.S., Hughes L.D. (2017). Understanding tamoxifen adherence in women with breast cancer: a qualitative study. Br J Health Psychol.

[16] Brett J., Boulton M., Fenlon D. (2018). Adjuvant endocrine therapy after breast cancer: a qualitative study of factors associated with adherence. Patient Prefer Adherence.

[17] Harrow A., Dryden R., McCowan C. (2014). A hard pill to swallow: a qualitative study of women's experiences of adjuvant endocrine therapy for breast cancer. BMJ Open.

[18] Ferreira A.R., Di Meglio A., Pistilli B. (2019). Differential impact of endocrine therapy and chemotherapy on quality of life of breast cancer survivors: a prospective patient-reported outcomes analysis. Ann Oncol.

[19] Franzoi M.A., Agostinetto E., Perachino M. (2021). Evidence-based approaches for the management of side-effects of adjuvant endocrine therapy in patients with breast cancer. Lancet Oncol.

[20] Hadji P., Blettner M., Harbeck N. (2013). The Patient's Anastrozole Compliance to Therapy (PACT) Program: a randomized, in-practice study on the impact of a standardized information program on persistence and compliance to adjuvant endocrine therapy in postmenopausal women with early breast cancer. Ann Oncol.

[21] Neven P., Markopoulos C., Tanner M. (2014). The impact of educational materials on compliance and persistence rates with adjuvant aromatase inhibitor treatment: first-year results from the Compliance of ARomatase Inhibitors AssessmenT in Daily practice through Educational approach (CARIATIDE) study. Breast.

[22] Yu K.D., Zhou Y., Liu G.Y. (2012). A prospective, multicenter, controlled, observational study to evaluate the efficacy of a patient support program in improving patients' persistence to adjuvant aromatase inhibitor medication for postmenopausal, early stage breast cancer. Breast Cancer Res Treat.

[23] Ziller V., Kyvernitakis I., Knöll D., Storch A., Hars O., Hadji P. (2013). Influence of a patient information program on adherence and persistence with an aromatase inhibitor in breast cancer treatment - the COMPAS study. BMC Cancer.

[24] Gray W.N., Netz M., McConville A., Fedele D., Wagoner S.T., Schaefer M.R. (2018). Medication adherence in pediatric asthma: a systematic review of the literature. Pediatr Pulmonol.

[25] Kelly M.P., Barker M. (2016). Why is changing health-related behaviour so difficult?. Publ Health.

[26] Shahin W., Kennedy G.A., Stupans I. (2019). The impact of personal and cultural beliefs on medication adherence of patients with chronic illnesses: a systematic review. Patient Prefer Adherence.

[27] Craig P., Dieppe P., Macintyre S., Michie S., Nazareth I., Petticrew M. (2008). Developing and evaluating complex interventions: the new Medical Research Council guidance. BMJ.

[28] O'Cathain A., Croot L., Duncan E. (2019). Guidance on how to develop complex interventions to improve health and healthcare. BMJ Open.

[29] Skivington K., Matthews L., Simpson S.A. (2021). Framework for the development and evaluation of complex interventions: gap analysis, workshop and consultation-informed update. Health Technol Assess.

[30] Frankland J., Brodie H., Cooke D. (2019). Follow-up care after treatment for prostate cancer: evaluation of a supported self-management and remote surveillance programme. BMC Cancer.

[31] Howell D., Mayer D.K., Fielding R. (2021). Management of cancer and health after the clinic visit: a call to action for self-management in cancer care. JNCI (J Natl Cancer Inst): J Natl Cancer Inst.

[32] Howell D.D. (2018). Supported self-management for cancer survivors to address long-term biopsychosocial consequences of cancer and treatment to optimize living well. Curr Opin Support Palliat Care.

[33] van der Hout A., van Uden-Kraan C.F., Holtmaat K. (2020). Role of eHealth application Oncokompas in supporting self-management of symptoms and health-related quality of life in cancer survivors: a randomised, controlled trial. Lancet Oncol.

[34] Boland L., Bennett K., Connolly D. (2018). Self-management interventions for cancer survivors: a systematic review. Support Care Cancer.

[35] Hammer M.J., Ercolano E.A., Wright F., Dickson V.V., Chyun D., Melkus G.D. (2015). Self-management for adult patients with cancer: an integrative review. Cancer Nurs.

[36] Kim S.H., Kim K., Mayer D.K. (2017). Self-management intervention for adult cancer survivors after treatment: a systematic review and meta-analysis. Oncol Nurs Forum.

[37] Chan A.H., De Simoni A., Wileman V. (2018). Digital interventions to improve adherence to maintenance medication in asthma. Cochrane Database Syst Rev.

[38] Chapman S., Sibelli A., St-Clair Jones A., Forbes A., Chater A., Horne R. (2020). Personalised adherence support for maintenance treatment of inflammatory bowel disease: a tailored digital intervention to change adherence-related beliefs and barriers. Journal of Crohn’s and Colitis.

[39] Toonders S.A.J., Westrienen PE van, Konings S., Nieboer M.E., Veenhof C., Pisters M.F. (2021). Patients' perspectives on the usability of a blended approach to an integrated intervention for patients with medically unexplained physical symptoms: mixed methods study. J Med Internet Res.

[40] Duncan E., O'Cathain A., Rousseau N. (2020). Guidance for reporting intervention development studies in health research (GUIDED): an evidence-based consensus study. BMJ Open.

[41] Hoffmann T.C., Glasziou P.P., Boutron I. (2014). Better reporting of interventions: template for intervention description and replication (TIDieR) checklist and guide. BMJ.

[42] Moon Z., Moss-Morris R., Hunter M.S., Norton S., Hughes L.D. (2019). Nonadherence to tamoxifen in breast cancer survivors: a 12 month longitudinal analysis. Health Psychol.

[43] Horne R., Cooper V., Wileman V., Chan A. (2019). Supporting adherence to medicines for long-term conditions. Eur Psychol.

[44] Leventhal H., Phillips L.A., Burns E. (2016). The Common-Sense Model of Self-Regulation (CSM): a dynamic framework for understanding illness self-management. J Behav Med.

[45] Leventhal H., Diefenbach M., Leventhal E.A. (1992). Illness cognition: using common sense to understand treatment adherence and affect cognition interactions. Cognit Ther Res.

[46] Horne R., Weinman J., Hankins M. (1999). The beliefs about medicines questionnaire: the development and evaluation of a new method for assessing the cognitive representation of medication. Psychol Health.

[47] Horne R., Chapman S.C.E., Parham R., Freemantle N., Forbes A., Cooper V. (2013). Understanding patients' adherence-related beliefs about medicines prescribed for long-term conditions: a meta-analytic review of the necessity-concerns framework. PLoS One.

[48] Cahir C., Bennett K., Dombrowski S.U. (2023). Informing interventions to improve uptake of adjuvant endocrine therapy in women with breast cancer: a theoretical-based examination of modifiable influences on non-adherence. Support Care Cancer.

[49] Horne R., Weinman J., Barber N., Elliot R., Morgan M. (2005).

[50] Nunes V., Neilson J., O'Flynn N. (2009).

[51] Moore G.F., Audrey S., Barker M. (2015). Process evaluation of complex interventions: Medical Research Council guidance. BMJ.

[52] Brett J., Boulton M., Watson E. (2018). Development of an e-health app to support women prescribed adjuvant endocrine therapy after treatment for breast cancer. Patient Prefer Adherence.

[53] Technology HOD Data and. Designing for accessibility. Accessed January 23, 2023. https://ukhomeoffice.github.io/accessibility-posters/.

[54] Design principles - NHS digital service manual. nhs.uk. Accessed January 23, 2023. https://service-manual.nhs.uk.

[55] Taylor S.J., Bogdan R., DeVault M. (2016). https://www.wiley.com/en-gb/Introduction+to+Qualitative+Research+Methods%3A+A+Guidebook+and+Resource%2C+4th+Edition-p-9781118767214.

[56] Bradbury K., Watts S., Arden-Close E., Yardley L., Lewith G. (2014). Developing digital interventions: a methodological guide. Evid base Compl Alternative Med.

[57] Abraham C., Michie S. (2008). A taxonomy of behavior change techniques used in interventions. Health Psychol.

[58] Horne R. (2003). The self-regulation of health and illness behaviour.

[59] Horne R., Weinman J. (2002). Self-regulation and self-management in asthma: exploring the role of illness perceptions and treatment beliefs in explaining non-adherence to preventer medication. Psychol Health.

[60] Moon Z., Moss-Morris R., Hunter M.S., Goodliffe S., Hughes L.D. (2019). Acceptability and feasibility of a self-management intervention for women prescribed tamoxifen. Health Educ J.

[61] Michie S., Richardson M., Johnston M. (2013). The behavior change technique taxonomy (v1) of 93 hierarchically clustered techniques: building an international consensus for the reporting of behavior change interventions. Ann Behav Med.

[62] Foster C. (2022). The need for quality self-management support in cancer care. BMJ Qual Saf.

[63] Rimmer B., Sharp L. (2021). On behalf of ways ahead study team. Implementation of self-management interventions in cancer survivors: why are we not there yet?. J Cancer Educ.

[64] Bandiera C., Skrabal Ross X., Cardoso E. (2022). Interventions to support adherence to oral anticancer therapies: research challenges, lessons learned, and strategies to overcome them from Australia and Switzerland. Support Care Cancer.

