# Supplementary material for: Development of the HT&Me intervention to support women with breast cancer to adhere to adjuvant endocrine therapy and improve quality of life
Source: Breast. 2023 May 27;70:32–40. doi: 10.1016/j.breast.2023.05.007 (PMC10382955; doi:10.1016/j.breast.2023.05.007)
Supplement: Multimedia component 1 [file mmc1.docx]

**Supplementary Material**

**Supplementary Material 1:**

*The GUIDED Checklist – a guideline for reporting for intervention development studies*

| **Item description** | **Page in manuscript where item is located** |
| --- | --- |
| 1. Report the context for which the intervention was developed. | Page 3 |
| 2. Report the purpose of the intervention development process. | Pages 3-4 |
| 3. Report the target population for the intervention development process. | Page 4 |
| 4. Report how any published intervention development approach contributed to the development process | Page 4 |
| 5. Report how evidence from different sources informed the intervention development process. | Pages 4-6 |
| 6. Report how/if published theory informed the intervention development process. | Page 5 |
| 7. Report any use of components from an existing intervention in the current intervention development process. | N/A |
| 8. Report any guiding principles, people or factors that were prioritised when making decisions during the intervention development process. | Pages 4-6 |
| 9. Report how stakeholders contributed to the intervention development process | Pages 4-6 |
| 10. Report how the intervention changed in content and format from the start of the intervention development process. | Pages 9-10 |
| 11. Report any changes to interventions required or likely to be required for subgroups. | Page 11 |
| 12. Report important uncertainties at the end of the intervention development process. | Page 11 |
| 13. Follow TIDieR guidance when describing the developed intervention. | Supplementary Material 2 |
| 14. Report the intervention development process in an open access format. | Open access publication of development paper |

**Supplementary Material 2:**

*The TIDieR (Template for Intervention Description and Replication) Checklist*

| **Item no.** | **Item** | **Where located** | |
| --- | --- | --- | --- |
|  |  | **Primary paper**  (Page or appendix number) | **Other** |
|  | **BRIEF NAME** | | |
| **1.** | Provide the name or a phrase that describes the intervention. | **Introduction** – page 4 |  |
|  | **WHY** | | |
| **2.** | Describe any rationale, theory, or goal of the elements essential to the intervention. | **Introduction** – pages 3-4  **Methods** – page 6 |  |
|  | **WHAT** | | |
| **3.** | Materials: Describe any physical or informational materials used in the intervention, including those provided to participants or used in intervention delivery or in training of intervention providers. Provide information on where the materials can be accessed (e.g. online appendix, URL). | *HT&Me Animation video*  **Results** – pages 7-8  *HT&Me web-app*  **Results** – pages 8-9  **Table 2** - pages 15-18  **Supplementary Material** **5**  *HT&Me Study Nurse training*  **Results** – page 8  *HT&Me Study Nurse consultation guide*  **Table 2** – page 15  **Table 4** – page 19  *Motivational ‘nudge’ email/text messages*  **Results** – page 9  **Table 2** – page 18 | HT&Me web-app (access approval is required)  <https://htandme.co.uk/> |
| **4.** | Procedures: Describe each of the procedures, activities, and/or processes used in the intervention, including any enabling or support activities. | *Optimisation studies*  **Methods** - pages 6-7  *Nurse consultations*  **Results** - page 8  *Sending ‘nudge’ messages*  **Results** - page 9 |  |
|  | **WHO PROVIDED** | | |
| **5.** | For each category of intervention provider (e.g. psychologist, nursing assistant), describe their expertise, background and any specific training given. | **Results** – page 8 |  |
|  | **HOW** | | |
| **6.** | Describe the modes of delivery (e.g. face-to-face or by some other mechanism, such as internet or telephone) of the intervention and whether it was provided individually or in a group. | **Results** – pages 7-8 |  |
|  | **WHERE** | | |
| **7.** | Describe the type(s) of location(s) where the intervention occurred, including any necessary infrastructure or relevant features. | **Results** – pages 7-8 |  |
| **8.** | **WHEN and HOW MUCH** | | |
|  | Describe the number of times the intervention was delivered and over what period of time including the number of sessions, their schedule, and their duration, intensity or dose | **Results** – pages 7-8 |  |
|  | **TAILORING** | | |
| **9.** | If the intervention was planned to be personalised, titrated or adapted, then describe what, why, when, and how. | **Results** – pages8 -9  *My Personal Support*  **Table 2** – page 17 |  |
|  | **MODIFICATIONS** | | |
| **10.** | If the intervention was modified during the course of the study, describe the changes (what, why, when, and how). | **Methods –** pages 6-7  **Results** – pages 9-10  *Optimisation study 1* **Supplementary Material 6**  *Optimisation study 2* **Supplementary Material 7** |  |
|  | **HOW WELL** | | |
| **11.** | Planned: If intervention adherence or fidelity was assessed, describe how and by whom, and if any strategies were used to maintain or improve fidelity, describe them. | **N/A** |  |
| **12.** | Actual: If intervention adherence or fidelity was assessed, describe the extent to which the intervention was delivered as planned. | **N/A** |  |

**Supplementary Material 3:**

*Participant characteristics of optimisation studies*

| **Characteristic** | **Optimisation study 1 (N = 20)** | **Optimisation study 2 (N = 15)** |
| --- | --- | --- |
| **Age** | | |
| 31-40 | 3 (15%) | 1 (6.6%) |
| 41-50 | 5 (25%) | 3 (20%) |
| 51-60 | 7 (35%) | 4 (26.6%) |
| 61-70 | 4 (20%) | 4 (26.6%) |
| 71-80 | 1 (5%) | 3 (20%) |
| **Education level** | | |
| A level (or equivalent) | 11 (55%) | 5 (33.3%) |
| Higher National Diploma (HND) | 1 (5%) | 0 |
| Undergraduate Degree (or equivalent) | 5 (25%) | 8 (53.3%) |
| Postgraduate Degree | 3 (15%) | 2 (13.3%) |
| **Ethnicity** | | |
| White British | 16 (80%) | 13 (86.6%) |
| White Other | 1 (5%) | 0 |
| Black British | 1 (5%) | 0 |
| Black African | 1 (5%) | 0 |
| Black Caribbean | 1 (5%) | 1 (6.6%) |
| Indian | 0 | 1 (6.6%) |
| **Employment** | | |
| Full time | 9 (45%) | 5 (33.3%) |
| Part-time | 3 (15%) | 3 (20%) |
| Retired | 5 (25%) | 6 (40%) |
| Unemployed | 2 (10%) | 1 (6.6%) |
| Student | 1 (5%) | 0 |

**Supplementary Material 4:**

**
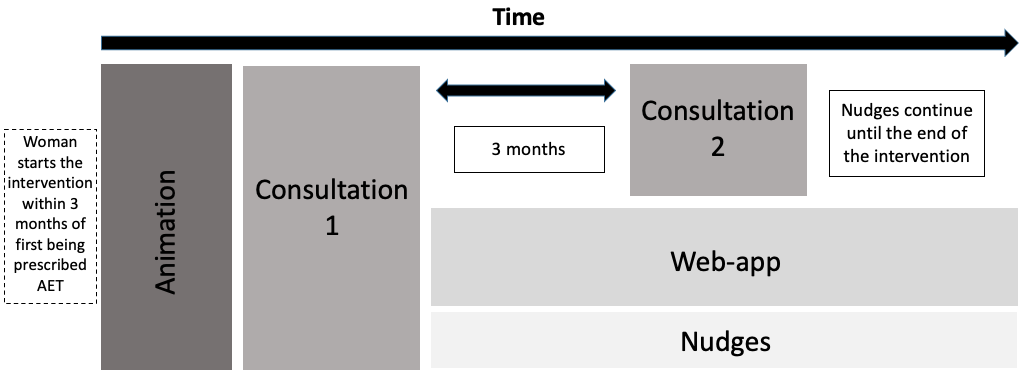
***An overview of the HT&Me intervention*

**Supplementary Material 5:**

*HT&Me web-app homepage*


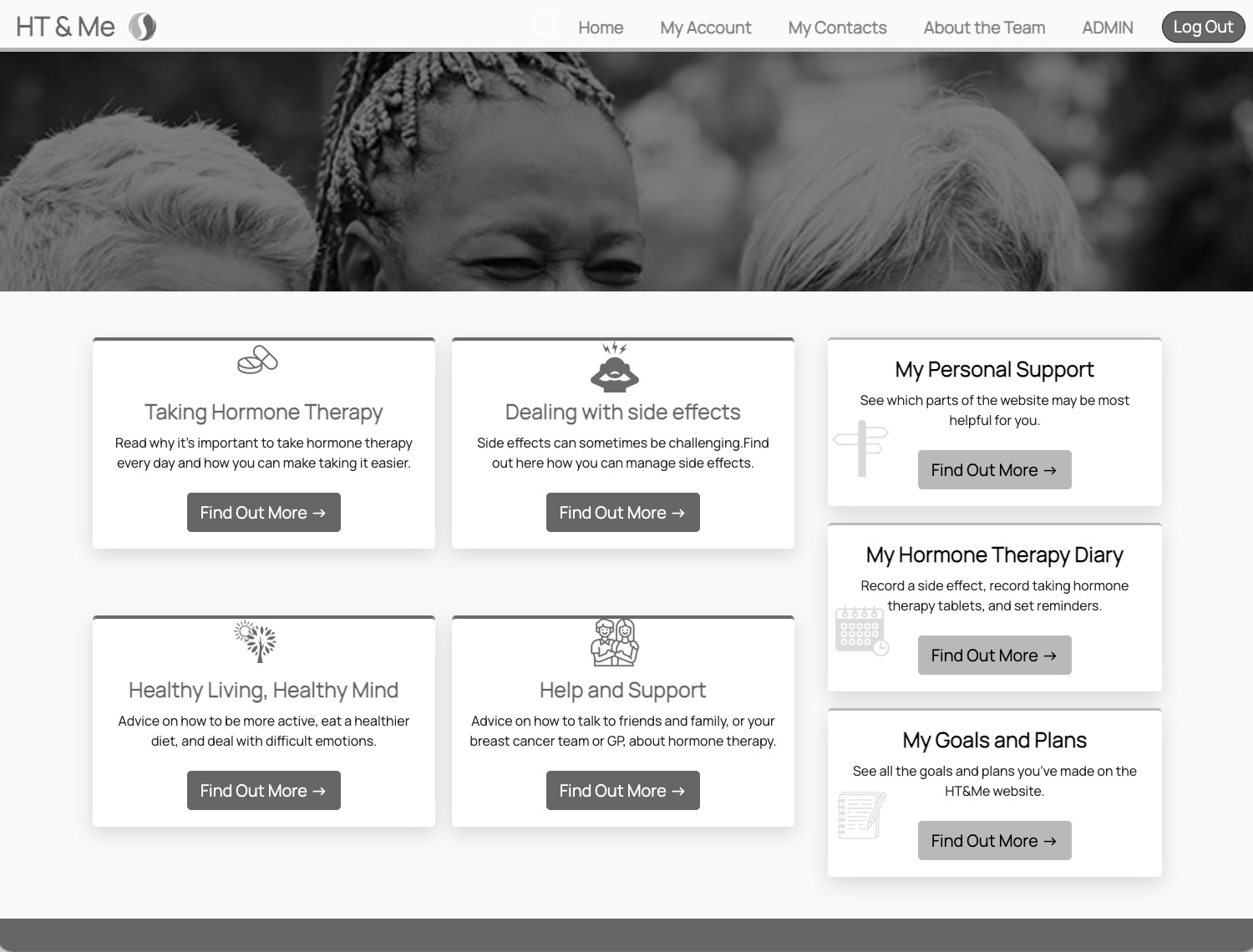


**Supplementary Material 6**:

An overview of positive feedback and key issues raised by participants in optimisation study 1 about the HT&Me animation and HT&Me web-app.

| **Positive feedback about HT&Me** | **Exemplar supporting quotation** | |
| --- | --- | --- |
| **Animation** | | |
| Animation video was informative and inclusive | *“…the inclusivity of it. I love that, all the imagery, the inclusivity is so important. That was brilliant. …*  *How it was explained about, again, oestrogen receptor. That was superb… I didn’t expect it to go into such depth of explanation about what Tamoxifen means and then what the AI, how it differentiates because that took me some time to understand that. It’s like well endocrine treatment, again, it’s one thing but it isn’t. So to have that explained in that way, I think that’s super important and that’s brilliant how that was done.”* (Cathy, 54yrs) | |
| **HT&Me: General comments** | | |
| Inclusion of quotes from other women | “*I think it’s really clear that you’ve spoken to people who have had cancer, who have been through all these things, and these are the feelings and the worries and the concerns that you have. It does put women who have had cancer in the centre of it.*” (Hanna, 44yrs) | |
| Clear language on web-app | *“I think it’s good. Like I said, it’s not patronising at all, I thought it was, yeah, it’s in more normal language to understand… It’s just, it’s in normal language, it’s not patronising, it’s not medical, and it’s not trying to deal with hundreds of different things.”* (Pip, 57yrs) | |
| Web-app content | “*‘I think it's... I don't think there's anything… I don't think there's anything you've missed in someone who's about to*  *start hormone therapy or on hormone therapy who is primary breast cancer. That's all. That’s all I would say. There’s nothing else anyone, I don’t think, would need.”* (Lucy, 49yrs) | |
| **Summary of Issue** | **Exemplar supporting quotation** | **Intervention modification(s)** |
| **Animation** | | |
| No video thumbnail | “*So, I’ve got the blank … So, I think that could easily be misconstrued as I’ll skip that part then.”*(Cathy, 54yrs) | - A thumbnail was created and inserted showing the title of the animation video |
| **HT&Me: General comments** | | |
| Confusion about website name | “*Sweet, why is it sweet because it isn’t sweet? It’s bloody awful.”* (Lucy, 49yrs) | - Web-app name was changed from SWEET (acronym of project title) to HT&Me |
| Younger women not feeling well represented on the website | “*It is quite an older person, well it’s perceived as being an older person’s thing so when you are a younger woman and you go through it, you can feel a little bit isolated. So, reading something from a younger woman as well would be good.”* (Kirsty, 35yrs) | - More quotations from younger women were inserted |
| **HT&Me: Taking Hormone Therapy** | | |
| Overwhelming amount of information in places | “*There’s a hell of a lot of info on here, I must say.*” (Joanne, 59yrs) | - Condensed information where possible. Where this could not be done, improvements were made to the presentation of information to make it more user-friendly - ‘Disclaimer’ was added to say that women may find it better to ‘dip in and out’ of content as and when is necessary. |
| Difficulty navigating through ‘tunnelled’ content, where users were required to click through pages in a sequence by clicking ‘Next’ or ‘Back’. This meant all women were presented with all content regardless of its relevance for them. | “W*hen it says back, does it sometimes need a bit that says back to homepage rather than just back? I’m not quite sure about that, you need to look at it. Sometimes it says back and I’m not sure whether I was going back to the homepage or going back one page.*” (Joy, 77yrs) | - Navigation throughout the web-app navigation was simplified to enable women to be able to ‘dip in and out’ of the content - Taking Hormone Therapy homepage layout was improved. |
| Clarity was needed regarding what to do if you occasionally forget to take a tablet. | “*I’m not sure what exactly ‘nearly time’ means? After how many hours it is fine to take a tablet when you forgot to take it. When should you wait for the next tablet?*” (Cathy, 54yrs) | - Consulted with clinical team and clarified content on the web-app accordingly. |
| **HT&Me: Dealing with side-effects** | | |
| Information in the Mood changes section was not specific enough. | “*It was bad you know, I was so irritable, everything played on my nerves, and I didn’t know how to deal with it. So what is it that I could have done? I don’t see it here … it’s all useful stuff. But I’m not really sure that it's all that specific.”* (Alice, 67yrs) | - Consulted with clinical team and added practical tips and tools for dealing with irritability and low mood. |
| Confusion that information about vaginal dryness and pain was under ‘Sexual Problems’. | “*It’s not just sexual, the major point isn’t the sexual part […] but it’s under your sexual heading. So, I don’t know, some people might not even look at that because maybe they’re not with anybody and it doesn’t bother them, or they’ve not got a partner at the moment.”* (Heidi, 53yrs) | - Changed the structure of the ‘Dealing with Side-effects’ section to create a new sub-section called ‘Vaginal dryness and pain’ separate to ‘Sexual Problems’. |
| **HT&Me: Healthy Living, Healthy Mind** | | |
| Missing information on ‘Being Active’ when in pain | “*So, it’s almost something like, ‘I’m in pain doing normal activities, can I be more active?’ Not necessarily running, but you just think, ‘Well, if it hurts to get out of bed in the morning, how can I go for a run?”* (Justine, 40yrs) | - Added more specialist information and tips for being physically active when pain in ‘Being Active’. |
| Lack of quotations from women in ‘Dealing with the Emotional Impact of Cancer’. | “*It would be quite nice to have somebody, other people talking about the effect it had and what they did about it*.” (Joy, 77yrs) | - Quotations from our patient advisory group were added to ‘Dealing with the Emotional Impact of Cancer’ to illustrate how they managed emotional difficulties surrounding cancer. |
| **HT&Me: Help & Support** | | |
| More information was needed about risk of cancer recurrence | “*There’s nothing really in there saying that, you know, cancer can come back and when it comes back it comes back as metastasis and it spreads to bones, brain, lungs […] It’s sort of missing out a taboo subject. And if you want to be factual then it has to be factual with everything because it's not always lumps and bumps, you know*.” (Lucy, 49yrs) | - Information added about risk of recurrence - Information added about help-seeking for pain/unusual symptoms |
| **HT&Me: My Hormone Therapy Diary** | | |
| Difficulty using ‘My Hormone Therapy Diary’ | “*I think why this is confusing is because most people won’t really know why this is the case and then you click this. So, you click on this, my hormone therapy diary. I think you need a little bit more info here […] so people know what they’re doing when they click the schedule reminder and also why they would want to fill out the hormone diary.”* (Elle, 34yrs) | - Instructions to use the diary were simplified using screenshots to illustrate - Diary was re-designed to be more user friendly e.g. improvements to how side-effects were presented on the graph. |
| **HT&Me: My Personal Support** | | |
| Participants did not notice the ‘scroll down’ function and therefore missed some of the questions about taking AET. | N/A – researcher observation | - Clarified instructions for completing ‘My Personal Support’. - Inserted a ‘submit responses’ button at the bottom of the page so that participants had to ‘scroll down’ and see all options to proceed. |

*Note*. Pseudonyms have been used to retain anonymity

**Supplementary Material 7**:

An overview of positive feedback and key issues raised by participants in optimisation study 2 about the HT&Me web-app

| **Positive feedback about HT&Me** | **Exemplar supporting quotation** | |
| --- | --- | --- |
| **Animation** | | |
| Informative and helpful | *“The videos are very interesting as well. They give you a lot of detail as well, which is some things you don't hear about. At the hospital you're just told this, this, this, and nothing else, no other information. So that has been helpful for me.”* (Susan, 60yrs) | |
| **HT&Me web-app: General comments** | | |
| Easy to navigate through and use | “*I didn’t really have a problem with the website at all, I thought it was very easy to use […] that’s what you want. And I think the fact it’s with big buttons, and things like that, it’s good. There’s less room for error, you know. Because I guess a lot of people would be in their 60s and upwards who get this diagnosis. So we’re the ones who need it to be simple.*” (Ann, 60yrs) | |
| Clear and accessible language | “*I thought it was good. I just thought it was a really clear, concise website […] I didn’t feel the language was confusing or difficult.*” (Helen, 64yrs) | |
| **HT&Me web-app tailored interactive elements** | | |
| Daily reminders to take AET were helpful | “*The thing that I probably like best about the website was the daily reminders. […] I did find it really useful. I liked the fact that you could select different options on how to get that reminder. So, I picked text message which is something that is likely to get my attention because it’s the only way my son communicates with me. So there's a thing on the phone and I set it every day and it actually did remind me once when I had forgotten to take it.*” (Claire, 51yrs) | |
| Usefulness of tailored ‘My Personal Support’ | “*It did direct me around where I needed to go […] it’s useful, yes. So, I’ve obviously put in about my side-effects and it’s redirecting me to the parts of the website that I need. So, I suppose that would be quite a good thing to do to start with, if you didn’t have time to sit down and go through the whole website. It could help you find your way to the bits that you need quite easily.*” (Sarah, 51yrs) | |
| ‘My Thoughts’ activity helpful to reframe negative thoughts | “…*completing your thoughts, I think that section was very, very beneficial to individuals. I think perhaps just having the release of putting the thought in there.*” (Michelle, 63yrs) | |
| ‘My Hormone Therapy Diary’ useful for recording side-effects | “*It’s simple. I mean you just put in what side-effects that you have or what it was and how it will affect your day-to-day life and you can put it up to a higher or a lower level of that day. And it gives you a graph of how you're feeling within that month and I think it's quite good*”, (Susan, 60yrs). | |
| Goal setting for healthy eating and being more active increased motivation | “*My goal was to eat more veg. I quite enjoyed that because it made me stop and think what I might realistically incorporate at the moment.*” (Maggie, 67yrs) | |
| **‘Nudge’ messages** | | |
| Frequency and content about right | “*I thought that was good, it was just a little prompt, and I was like, “Oh, yes, I can do that now,” or, “I’ll do that later.” So that was good, yeah.*” (Tracy, 47yrs) | |
| **Issue summary** | **Exemplar supporting quotation** | **Intervention modification(s)** |
| **HT&Me: General comments** | | |
| HT&Me web-app not well optimised for use on mobile phone devices | “*[On laptop] it’s much easier to enter data, [on the phone] it didn’t quite fit sometimes on the screen, I think it’s because it’s not an app […] I didn’t really try much else on my phone because it just wasn’t user-friendly the screen really, it’s too small on my phone.”* (Sarah, 51yrs) | - Discussed with web design team to optimise use of HT&Me on mobile devices (e.g. changing font size and layout on mobiles to present content so it fits the screen). |
| When using HT&Me on a mobile phone, some pages appeared very long, which made it difficult to navigate through pages (‘back’ buttons are at top or bottom of pages) and get back to the homepage. | “*[When using a calendar in the diary] Yeah, on your phone it’s long... I think it’s more confusing on your phone to look at it than it is on your desktop.”* (Gabrielle, 46yrs) | - Added a ‘homepage’ button on the top banner of the web-app so participants can easily navigate back to the homepage at any point. |
| The text boxes to complete the CBT style ‘My Thoughts exercises were too small making it difficult for participants to revisit what they had written previously. | “*I fill out the critical negative thoughts and then a supportive neutral thought and I deal with my thoughts activities, manage change etc […] I found that I couldn’t re-read what I’d written. I couldn’t find a mechanism for just… just clicking on it to see what you’ve written, do you see what I mean?*” (Maggie, 67yrs) | - Increased the size of the text boxes. - Fill in boxes expand to accommodate and display the whole text. |
| Option to connect more with other breast cancer patients (e.g., to share their experiences of dealing with different side-effects) was not prominent. | “*So, maybe that ‘Breast Cancer Now’ forum just needs to be more prominent on your web […] you know, speak to find out stories, because I think that’s the one that is a really good one, isn’t it?* (Sarah, 51yrs) | - Made more prominent links to the Breast Cancer Now Patient Forum on the ‘Dealing with Side-effects’ and ‘Help and Support’ homepages. |
| **HT&Me: Taking Hormone Therapy** | | |
| Text bolding was confusing in the Taking Hormone Therapy section, and mistaken for links to other pages. | “*I found that a bit confusing, because some of it was in bold and I thought that was what you clicked on to find out more about it. Then you actually click further down but that one doesn’t state that, and actually on other sections, it does say click below for more information or something, but on that first page, I mean I did find it, but it was bit like, do I click on this bold or how do I find this?*” (Helen, 64yrs) | - Added a sentence to clarify that further information can be found by clicking the buttons on that page. - Reviewed the text in bold-face to maximise clarity. |
| Missing information about how AET differs to hormone replacement therapy. | “*Oh, it doesn’t mention actually, the website, the difference between…HRT is not mentioned. It’s funny, I get when I’m not thinking about it properly. My sister was talking about HRT the other day and I was like, “Yes, I am on that,” and I went, “No, hang on, I’m on the opposite of that. I’m on hormone therapy not hormone replacement therapy. Those two things are very different*.” (Karen, 44yrs) | - Added information about the differences between AET and hormone replacement therapy in the ‘Taking Hormone Therapy’ section. |
| **HT&Me: Dealing with Side-effects** | | |
| Missing information and advice for the experience and management of chills after getting hot flushes. | “*I had side-effects that weren’t on there […] my main one would be hot flushes and then after I have a hot flush, I get really cold as well, I get chills and I think that’s something I’ve read other women experience because you lose all the heat and then you’re cold. So, for me, the chills in a way, I dislike them more than the hot flush in a way because you always have to have loads of layers because you get cold and then have to put them back on*.” (Sarah, 51yrs) | - Consulted with clinical team and added text in the hot flushes section with advice on how to manage chills that can occur after hot flushes. |
| **HT&Me: Healthy Living, Healthy Mind** | | |
| Examples of physical activity in the ‘Being Active’ section were too advanced. | “*In the healthy living, healthy mind, I couldn’t find it … Perhaps something a little more of a beginner level [laughter], that’s slightly advanced, standing on one leg. … Something less advanced, I don’t know that many people who can stand on one leg without falling over, older people anyway.*” (Maggie, 67yrs) | - Included less advanced examples of ways to be active in the ‘Being Active’ section (e.g. added links to seated activities on the NHS website). |
| **HT&Me: Help and Support** | | |
| Section about rights at work after having breast cancer in the ‘Help and Support’ section was not clear. | “*I don’t know but coming out of the section, I was not sure what are my rights at work, do you know what I mean, I read through it, but the blank portion of my head is still blank. It just talks a little about reasonable adjustments which I’m aware of, is there anything else, I’m not sure, it’s something that I need to know.*” (Priya, 36yrs) | - Reviewed this section to make it clearer. |
| **HT&Me: My Goals and Plans** | | |
| Instructions for making a plan to take AET were not clear, meaning participants did not see the value in doing so. | *“I don’t know what I need to do, I mean there is just one pill that I need to take every day, what exactly is the plan that I need to do?”* (Priya, 36rs) | - Made the text outlining the value of making a plan to take AET every day clearer. - Made edits to the Study Nurse consultation guides to ensure HT&Me Study Nurses would emphasise the value in making a plan to take AET and completing the other HT&Me web-app interactive elements in the nurse consultations. |
| **HT&Me: My Hormone Therapy Diary** | | |
| Suggestion that it would be helpful to record the brand of hormone therapy tablets in ‘My Hormone Therapy Diary’ to help with the self-management of side-effects. It was suggested to display this information on the graph (or in the calendar). | “*A good thing to monitor as well is the brands of tablets that people are taking. The brands make a difference too. I don’t know why they do, but I’ve had some random, really awful, with me, and I remember my cancer nurse saying to me that brands can really affect people in a different way. So, I think it would be a good thing to have a note on your symptoms, also to note the brand you’re taking at that time because that can also have an effect on your side-effects*.” (Gabrielle, 46yrs) | - Added an option to record the brand (or change of brand) of AET in ‘My Hormone Therapy Diary’. - Added the ability to indicate this on the side-effects graph so that women can track and display their side-effects before and after a medication change. |
| An error with the function to record the number of hot flushes for a given day. | “*I think my side-effects just show a line of constant hot flushes. There was a bit that came up once that said, “How many hot flushes are you having a day?” Then, I never saw that bit again*.” (Gabrielle, 46yrs) | - Corrected an error causing this problem. |
| Adding multiple side-effects for a given day was not possible at the same time, and therefore not user-friendly. | “*I didn’t find it that friendly for putting on more side-effects*.” (Sarah, 51yrs) | - Improved the function to record multiple side-effects at one time, by ticking a box next to side-effects they want to add to the diary for a given day rather than selecting from the drop down menu. |

*Note*. Pseudonyms have been used to retain anonymity
